# Serologic evidence of mpox virus exposure among people living with human immunodeficiency virus in Thailand predating clinical recognition during the 2022 outbreak

**DOI:** 10.1016/j.ijregi.2026.100997

**Published:** 2026-09-04

**Authors:** Edgie-Mark A. Co, Paramate Promnarate, Michelle Imbach, Kristina Peachman, Camila Macedo Cincotta, Nittaya Phanuphak, Lydie Trautmann, Sandhya Vasan, Eric C. Garges

**Affiliations:** 1 Department of Retrovirology, Armed Forces Research Institute of Medical Sciences, Bangkok, Thailand; 2 Global Infectious Diseases, Henry Jackson Foundation, Bethesda, MD, USA; 3 Diagnostics and Countermeasures Branch, Walter Reed Army Institute of Research, Silver Spring, MD, USA; 4 Institute of HIV Research and Innovation, Unit 1109-1116, Bangkok, Thailand

**Keywords:** Mpox, HIV, Serosurveillance, Thailand

## Abstract

**Objectives:**

Mpox (also known as *monkeypox*) can cause significant morbidity in immunocompromised individuals, as evidenced in multiple recent outbreaks in non-endemic countries. We assessed orthopoxvirus immunoglobulin G seroprevalence in an at-risk Thai cohort to identify prior exposure and associated clinical and behavioral features.

**Methods:**

Archived 2022 specimens from an acute human immunodeficiency virus cohort were selected based on risk criteria, including multiple partners, a history of sexually transmitted infection, or drug use. Samples were tested using the Meso Scale Discovery V-PLEX Orthopoxvirus immunoglobulin G assay (Meso Scale Discovery, Rockville, MD, USA). Demographic, clinical, and behavioral data were analyzed using Mann–Whitney *U* and Fisher’s exact tests.

**Results:**

Of 491 participants, 5.5% (27/491) were seropositive. Seropositivity was detected as early as January 2022, predating Thailand’s first clinical case (July 2022) and wastewater detection (June 2022). Only 7.4% (2/27) of seropositive participants had abnormal physical examinations. Illicit drug use (74.1%) and meeting multiple behavioral risk criteria were significantly associated with seropositivity (*P* < 0.001 and *P* = 0.0317, respectively). No significant differences were found regarding CD4 counts, human immunodeficiency virus viral load suppression, or concurrent STIs.

**Conclusion:**

Unrecognized, paucisymptomatic mpox transmission occurred in Thailand months before official detection. These findings highlight the utility of serologic surveillance within established high-risk cohorts for the early detection of emerging infectious threats.

## Highlights

• Mpox seroprevalence was 5.5% in a high-risk cohort of people living with human immunodeficiency virus in Bangkok during 2022.
• Seropositivity was detected as early as January 2022, predating clinical reports.
• Findings suggest unrecognized transmission occurred months before the official outbreak.
• Illicit drug use and having multiple behavioral risk criteria were significantly associated with orthopoxvirus seropositivity.
• Serosurveillance in existing cohorts is a vital tool for emerging disease detection.

## Introduction

Monkeypox virus is an enveloped double-stranded DNA virus of the *Orthopoxvirus* genus comprising two distinct clades. Mpox disease results in a wide spectrum of clinical severity, particularly among individuals with immunocompromizing conditions. In 2022, an outbreak of mpox clade IIb occurred in non-endemic countries, disproportionately affecting specific sexual networks and populations at increased risk for sexually transmitted infections (STIs). It was subsequently declared a Public Health Emergency of International Concern (PHEIC) by the World Health Organization (WHO). Subsequently, mpox became a priority for public health and biodefense surveillance due to concerns regarding international spread and severe disease in vulnerable populations. Although there was a decline in cases following the 2022 outbreak, a subsequent PHEIC was declared in August 2024 due to the rise of the more severe clade 1b and was only recently rescinded in September 2025 [1]. These dynamics highlight the limitations of case-based surveillance for detecting infections with mild, atypical, or asymptomatic presentations. Although case counts have declined in many regions, mpox remains a Risk Group 3 human pathogen according to the WHO classification due to its significant potential to cause severe disease and pandemics [2]. It also remains an African continental emergency as indicated by the Africa Centres for Disease Control and Prevention [3]. Mpox resurgences and continued transmission have underscored the need for improved surveillance strategies, particularly in regions where routine mpox diagnostics are limited. Serologic surveillance has emerged as a useful complementary approach for identifying prior exposure and estimating the burden of undiagnosed infection, especially in populations at elevated risk [4,5].

People living with human immunodeficiency virus (PLHIV) represent a population of particular epidemiologic interest for mpox surveillance. A recent meta-analysis showed that mpox and human immunodeficiency virus (HIV) co-infections result in higher mortality rates than are observed in people with mpox without HIV co-infection [6]. The same publication also indicated that decreased CD4 counts significantly affect the risk of hospitalization and death. Even among clinically stable PLHIV receiving antiretroviral therapy, shared behavioral and network-level risk factors may increase exposure to mpox during outbreaks.

In Bangkok, Thailand, the RV254 cohort is a well-characterized longitudinal study designed to identify acute HIV infection among individuals at high risk for HIV acquisition and also serves as a platform for recruitment into interventional cure studies [[7], [8], [9]]. This cohort mainly comprises men who have sex with men (MSM). As part of holistic HIV care, the study also conducts basic hematological and chemistry diagnostics and testing for other STIs such as chlamydia, gonorrhea, syphilis, and hepatitis viruses, while collecting behavioral and demographic data. The cohort also maintains a longitudinal biorepository of serum, plasma, and other biospecimens collected from the time of acute HIV diagnosis. In addition, these collected samples allow retrospective laboratory testing to evaluate demographic and behavioral risk factors during HIV infection [10], including detection of novel and emerging infectious diseases in a unique at-risk population. Thus, RV254 provided an opportunity to retrospectively assess orthopoxvirus seroprevalence during the 2022 mpox outbreak period in Thailand and to explore demographic and behavioral correlates of seropositivity.

This analysis aims to contribute regional data to the growing literature on mpox seroepidemiology and to inform the potential role of serologic surveillance in identifying undetected transmission in high-risk populations.

## Materials and methods

### Sample selection

We performed a cross-sectional serologic analysis using archived serum samples. Specimens collected in 2022 were selected from the RV254 repository using predefined risk-based criteria. Eligible participants met at least one of the following criteria at the time of sample collection: ≥10 sexual partners in the past year, or ≥3 sexual partners in the past 3 months; laboratory-confirmed STI positivity in the past month, or a history of illicit drug use. For STI testing, positivity was defined as having a positive or reactive result at any anatomical site, including pharyngeal, anal, or urine samples. If multiple samples were available from a given participant during 2022, only the earliest sample was selected for subsequent laboratory testing to avoid overrepresentation of individuals with repeated sampling. This selection strategy was designed to enrich for individuals at higher risk of mpox exposure; therefore, the resulting sample represents a convenience sample of PLHIV at higher risk and is not intended to be representative of the broader PLHIV population in Thailand.

### Laboratory methods

Orthopoxvirus seropositivity was determined using the precommercially released V-PLEX Orthopoxvirus Serology Kit Panel 1 (immunoglobulin G [IgG]) with the Meso Scale Discovery (MSD) S600 instrument (Meso Scale Discovery, Rockville, MD, USA). Baseline negative Monkeypox virus E8L antigen levels were characterized using institutional review board-approved samples obtained from the RV254 cohort, specifically from the 2014-2016 period before the mpox outbreak in Thailand; analytical sensitivity, specificity, cutoff thresholds, and immune background were characterized as described previously [11].

### Data extraction

After laboratory testing and subsequent identification of mpox-seropositive participants from the 2022 testing pool, the following data were abstracted from clinical research forms (CRFs) from the time of sample collection: physical examination findings, HIV viral load, CD4 count, white blood cell count (including differentials), platelet count, rapid plasma reagin (RPR) titers, current antiretroviral therapy (ART) and number of missed ART doses, if any. Behavioral data, including reported sexual partners and substance use, were obtained from routinely collected cohort questionnaires administered at study visits.

### Statistical analysis

Descriptive statistics were used to summarize demographic, clinical, and behavioral characteristics of the overall analytic sample and by serologic status. Continuous variables were summarized using medians and interquartile ranges, and categorical variables were summarized using frequencies and percentages. Statistical analysis was conducted using GraphPad Prism v10.6 software (GraphPad Software, San Diego, CA, USA). Continuous variables were compared using the Mann–Whitney *U* test and categorical variables using Fisher’s exact test. Due to the exploratory nature of the analysis and the small number of seropositive participants, no multivariable modeling was performed. Differences were considered significant if *P* < 0.05.

## Results

**Figure 1:**
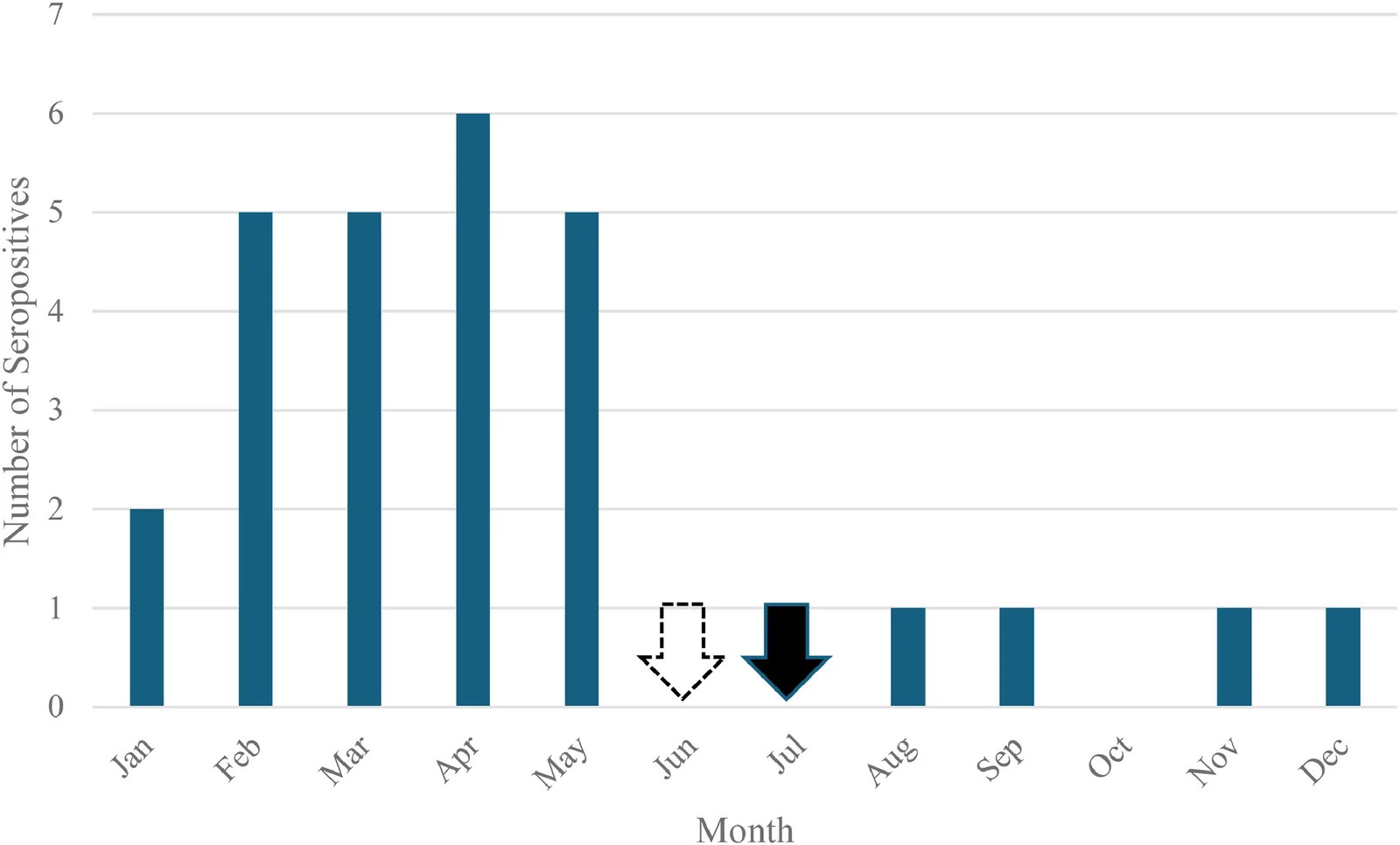
Number of mpox-seropositive participants per month. The block arrow denotes the month when the fir^st^ positive case was reported in Thailand (July 2022), whereas the dashed arrow indicates the month of wastewater identification (June 2022).

A total of 491 archived serum samples collected in 2022 from unique RV254 participants meeting the risk-based selection criteria were included in the analysis. Demographic and clinical characteristics of the tested cohort and seropositive participants are summarized in Table 1. Among the tested cohort, the median participant age at sample collection was 33 years (interquartile range, 29-38 years). Of these participants, 113 (23%) had ≥3 sex partners, 23 (4.7%) were gonorrhea positive, 30 (6.1%) were chlamydia positive (any site), and 204 (41.5%) were RPR reactive. Orthopoxvirus IgG testing showed that 5.5% (27/491) of the cohort were positive for mpox antibodies. Among seropositive participants, 85.2% (23/27) were from the first 5 months of 2022, with the earliest identified in January and new seropositive detections leveled off to about 1 per month by December 2022 (Figure 1). Approximately 80% of seropositive specimens were collected before the first clinically recognized case or wastewater detection reported in Thailand. Table 1 shows the clinical characteristics of seropositive participants. Among seropositive participants, 2/27 had abnormal physical examination results. An abnormality noted in one of the participants was “penile chancre,” while the other had “Chancroid 3mm +erythema, tender to palpation.” Both of these participants were RPR reactive at a titer of 1:2. Routine laboratory parameters, including white blood cell counts, differentials, and platelet counts, were within normal reference ranges for most seropositive participants. All seropositive participants were receiving antiretroviral therapy at the time of sample collection with well-suppressed HIV plasma viral loads below 50 copies/mL. Among all tested participants who reported missing ART doses over a 12-month period prior to clinic visits, the average number of days missed was 1.875 days.

**Table 1:** Demographic and clinical characteristics of the tested RV254 cohort and mpox-seropositive participants, 2022.

Variable	Total (N = 491)	Seropositive participants (N = 27)	*P*-value
Age (y), median (IQR)	33 (29-38)	32 (29.5-37)	0.8
Sex partners ≥3, *n* (%)	113 (23.0%)	7 (25.9%)	0.91
GC-positive, *n* (%)	23 (4.7%)	0 (0%)	0.62
CT-positive, *n* (%)	30 (6.1%)	1 (3.7%)	1.00
RPR-positive, *n* (%)	204 (41.5%)	13 (48.1%)	0.63
Drug use, *n* (%)	44 (9.0%)	20 (74.1%)	<0.001
Risk factors ≤1, *n* (%)	412 (95.8%)	18 (4.2%)	0.0317
≥2	79 (89.8%)	9 (10.2%)	–

*P* < 0.05 was considered statistically significant.

Abbreviations: CT, chlamydia; GC, gonorrhea; IQR, interquartile range; RPR, rapid plasma reagin.

When compared with seronegative participants, seropositive individuals were more likely to report illicit drug use and to meet at least two predefined behavioral risk criteria (Table 1). No statistically significant differences were observed between seropositive and seronegative participants with respect to CD4 cell counts, HIV viral load suppression, or the presence of concurrent STIs. Finally, 22 (81.5%) of the seropositive participants were younger than 42 years at the time of sample collection. The five participants aged 42 years or older ranged in age from 42-70 years and were born before smallpox vaccination was discontinued in Thailand. None of these participants had documentation of smallpox vaccination during CRF review.

## Discussion

In this cross-sectional serologic analysis of archived specimens collected in 2022, we identified orthopoxvirus IgG seropositivity suggestive of prior mpox exposure in 5.5% of a risk-enriched cohort of PLHIV in Thailand. Our results also indicate seropositivity at dates earlier than detection by other methods. Specifically, the first clinical case in Thailand was not reported until July 21, 2022; the case was identified in a Nigerian man traveling to Phuket who was reported to have been ill for about 1 week before presenting for clinical care [12]. In another report, mpox DNA was identified in wastewater collected around Bangkok in June 2022 [13]. This group tested wastewater from several locations in Bangkok using a previous severe acute respiratory syndrome coronavirus 2 wastewater surveillance program from May to August 2022 [14]. Hence, our findings suggest potential orthopoxvirus seropositivity consistent with prior mpox exposure as early as January 2022. This timing and transmission kinetics align with observations in the United States and Europe, where delays in overt clinical presentation were noted [15,16]. Costello et al. [15] described a case with an absence of overt human contact, while Ianache et al. [16] noted a more prolonged, sporadic paucisymptomatic spread. While these patterns are compatible with earlier, unrecognized mpox exposure than was suggested by clinically reported cases in Thailand, serologic data alone cannot establish the timing, location, or mode of infection. Travel restrictions in Thailand were lifted incrementally around the middle of 2021, which may have allowed asymptomatic or paucisymptomatic individuals to enter the country. Although the region is not known to have autochthonous pox virus endemicity [17], another possible reason for apparent orthopoxvirus seroconversion is a history of smallpox vaccination, which is known to cross-react with antibodies within the MSD assay [18,19]. A small proportion of participants (18.5%) were within the age range for having received smallpox vaccination, but we could not confirm this during CRF review.

Data from our cohort show an initial peak in seropositivity in the first part of 2022, with a decrease in seropositive results by the end of the year. This pattern is similar to that reported by the Thailand Ministry of Public Health during that time [20]. This temporal distribution of seropositivity in our cohort—concentrated predominantly in the first 5 months of 2022, followed by a plateau—warrants careful consideration. While low-level, unrecognized transmission likely occurred prior to global and national clinical recognition, the subsequent stabilization of new seropositive detections in late 2022 despite the absence of widespread vaccination may reflect rapid behavioral adaptation within key populations. Following international alerts in May 2022 and the WHO PHEIC declaration in July 2022, civil society organizations, academics, community-based clinics, and the Thai Ministry of Public Health initiated targeted health communication and risk-reduction campaigns aimed at MSM and high-risk networks in urban centers such as Bangkok [21,22]. Similar to observational findings in the United States and Europe [[23], [24], [25], [26]], early public health messaging led to proactive risk mitigation—including reductions in one-off sexual partners, increased symptom vigilance, and temporary modifications in sexual networking behaviors—prior to or in tandem with vaccine availability. Furthermore, because our cohort selection required active study engagement and routine STI screening [9], participants may also have benefited from early risk-reduction counseling during cohort visits, potentially contributing to a lower incidence of new exposures during the latter half of the year. Although mpox vaccine (JYNNEOS/IMVANEX/IMVAMUNE) is available in many other countries, it is not yet fully approved by the Thailand Food and Drug Administration for general use in the country. Vaccine supply is currently limited and prioritized for high-risk groups such as health care workers and close contacts of confirmed cases [27]. As of this writing, about 10 of the RV254 participants have received the JYNNEOS vaccine since the outbreak began (Promsena, personal communication).

When we compared the characteristics of the tested cohort and seropositive participants, participants reporting drug use and multiple stated risk factors were significantly more likely to be seropositive. These findings are consistent with other comparable studies on risk behaviors associated with mpox infection [28,29]. Interestingly, STI co-infections were not as strongly associated with mpox seropositivity in our cohort as expected. This cohort also has a high prevalence of RPR (syphilis) positivity. Syphilis can have a clinical presentation similar to mpox, and the high RPR prevalence is consistent with that seen in other studies of undetected and undiagnosed mpox infection [28,29]. Both factors underscore the potential areas for intervention to influence and prevent disease transmission. Unfortunately, the CRFs do not specify the types of substances (hallucinogen, stimulant, etc.), or participants did not report the type of substance used, which may be relevant to the types of potential interventions proposed.

Among seropositive participants in our cohort, we identified a few interesting clinical characteristics. Almost all tested participants had normal physical examination results at the time of their study sample collection. Of the two participants with abnormal physical examinations, both were RPR reactive (titer 1:2); one presented with a penile chancre characteristic of primary syphilis, while the other exhibited a localized rash and papule consistent with secondary syphilis or concurrent STI presentation. Unfortunately, no additional data on follow-up mpox testing are available for these participants. Similarly, nonspecific laboratory markers for infection such as white blood cell count were all within normal limits. With regard to their HIV status, all had normal CD4 counts, were receiving appropriate antiretroviral treatment per the Thailand Ministry of Public Health [30], and were adherent to ART (i.e. none had missed any doses). Missing >1 dose is considered a risk factor for developing HIV resistance [31] and thus nonadherence may potentially be a risk factor for mpox acquisition. Since none of the seropositive participants reported missing any ART doses, immunosuppression is unlikely to explain the observed transmission patterns within this cohort at the time of mpox testing. Hence, we can surmise that the transmission pattern and kinetics are likely similar to the population without HIV, given the high rate of silent transmission in this functionally immunocompetent population.

This study has some important limitations. First, the analysis was conducted in a risk-enriched convenience sample of the research cohort and is not an accurate representation of the general population or all PLHIV in Thailand. Additionally, the retrospective nature of the study and thus the potential for recall bias also limit clinical detection of mpox. At the time, the cohort survey and testing parameters were not designed to capture mpox cases, which also restricted data capture of other potentially relevant risk factors. Also, serologic assays for orthopoxviruses are subject to non-differential misclassification due to cross-reactivity with prior smallpox vaccination or exposure to other orthopoxviruses, which may bias prevalence estimates in either direction. We were unable to measure this impact due to the unavailability of smallpox vaccination data in study case report forms. Fortunately, 416 (85%) of the tested participants were born after smallpox vaccination was discontinued in Thailand (in 1980). Hence, we expect that any seroconversions identified in this study are more likely to reflect new infections than remote smallpox vaccination. Finally, the cross-sectional nature of the analysis precludes inference regarding incidence or precise timing of infection.

Despite these limitations, this study demonstrates the utility of serologic surveillance in well-characterized cohorts for identifying previously unrecognized mpox exposure. Established population-based cohorts have been used previously to identify and track disease outbreaks [32,33]. However, most are typically designed to track a single disease or diseases with similar manifestations. Immunocompetent populations often exhibit milder or subclinical presentations, facilitating undetected transmission. In this study, we highlight the value of serosurveillance within a high-risk cohort by identifying asymptomatic infections that can fuel future outbreaks. Owing to their increased exposure risk and greater likelihood of clinically apparent disease, these populations can function as effective sentinel groups for the early detection of novel and emerging infectious threats, including mpox.

In conclusion, our findings illustrate a modest mpox seroprevalence in our RV254 cohort in a population of PLHIV that is consistent with what has been reported in the literature. Asymptomatic or paucisymptomatic infection is a transmission concern because of the limited number and overlap of compatible signs and symptoms and thus presents a public health risk. To better understand the extent of mpox transmission within at-risk populations, a greater understanding of transmission dynamics and risk factors associated with infection is needed. These findings highlight the potential role of existing cohorts and seroepidemiologic approaches in improving early detection of emerging infections and informing public health response in high-risk populations.

## CRediT authorship contribution statement

**Edgie-Mark A. Co:** Writing – review & editing, Writing – original draft, Methodology, Investigation, Formal analysis, Conceptualization. **Paramate Promnarate:** Investigation. **Michelle Imbach:** Writing – review & editing, Investigation. **Kristina Peachman:** Writing – review & editing, Investigation. **Camila Macedo Cincotta:** Writing – review & editing, Investigation. **Nittaya Phanuphak:** Writing – review & editing. **Lydie Trautmann:** Writing – review & editing. **Sandhya Vasan:** Writing – review & editing. **Eric C. Garges:** Writing – review & editing, Supervision, Resources.

## Declaration of competing interest

The authors have no competing interests to declare.
